# Predictors and long-term outcomes of heart block after transcatheter device closure of perimembranous ventricular septal defect

**DOI:** 10.3389/fcvm.2022.1041852

**Published:** 2022-10-25

**Authors:** Diandong Jiang, Simiao Zhang, Yuxin Zhang, Jianli Lv, Yingchun Yi, Jing Wang, Yan Wang, Xiaofei Yang, Jianjun Zhang, Bo Han

**Affiliations:** ^1^Department of Pediatric Cardiology, Shandong Provincial Hospital Affiliated to Shandong First Medical University, Jinan, China; ^2^Department of Pediatrics, Shandong Provincial Hospital, Shandong First Medical University and Shandong Academy of Medical Sciences, Jinan, China

**Keywords:** heart block, perimembranous ventricular septal defect, transcatheter closure, predictors, outcomes

## Abstract

**Background:**

Heart block is the most common and concerning complication associated with transcatheter device closure of perimembranous ventricular septal defect (pmVSD) and its occurrence remains a great challenge for device closure.

**Methods:**

Between June 2002 and June 2020, 1076 pediatric patients with pmVSD, who successfully underwent transcatheter device closure in our center, were enrolled in this cohort study, with a median follow-up of 64 months (range: 1 to 19 years).

**Results:**

Of 1076 patients, 234 (21.8%) developed postprocedural heart block, with right bundle branch block being the most common (74.8%), followed by left bundle branch block (16.2%), and atrioventricular block (5.6%). Complete atrioventricular block occurred in 5 cases, including 3 cases with permanent pacemaker implantation, 1 case with recovery to normal sinus rhythm, and 1 case with sudden cardiac death. Most patients (97.9%) developed heart block within 1 week of procedure. Finally, 138 cases returned to normal cardiac conduction. Multivariate logistic regression revealed that thin-waist occluders (odds ratio [OR]: 1.759; 95% confidence interval [CI]: 1.023 to 3.022; *P* = 0.041), and oversized devices (OR: 1.809; 95% CI: 1.322 to 2.476; *P* < 0.001) were independently associated with occurrence of postprocedural heart block. Moreover, heart block was less likely to occur when the left disk of occluder was placed within the aneurysmal tissue (OR: 0.568; 95% CI: 0.348 to 0.928; *P* = 0.024).

**Conclusion:**

The outcome of postprocedural heart block is favorable in most cases. Oversized devices and thin-waist occluders should be avoided. Placement of the left disk of the device should into the aneurysmal tissue is highly recommended.

## Introduction

Ventricular septal defect (VSD) is one of the common types of congenital heart diseases (CHD), with perimembranous ventricular septal defect (pmVSD) subtype being the most frequent (70% of cases) (1). With recent developments in closure devices and interventional techniques, transcatheter device closure of pmVSD, as an alternative to conventional surgical repair, has shown promising results (2–4). Moreover, percutaneous device closure of pmVSD often leads to adverse heart events like bundle branch block (BBB) and atrioventricular block (AVB). Notably, earlier investigations were primarily focused on complete AVB (cAVB) complications (5, 6). Hence, reports on the diagnosis and treatments of other types of heart block, such as left and right BBBs are scarce. A growing body of evidence suggests postprocedural complete left bundle branch block (CLBBB) as a serious complication observed in pmVSD surgery (7). CLBBB can lead to abnormal left ventricular (LV) contraction which can induce progressive LV remodeling and heart failure (HF) (8). Under certain conditions, postprocedural heart block may recur or even progress to serious adverse events during the follow-up (6, 7). Nevertheless, the exact underlying mechanism of heart block is not well understood. Moreover, the etiological risk factors and treatment outcomes of heart block are not well defined for the pmVSD closure intervention in pediatric patients. Therefore, the present study was designed to investigate the rate of incidence, risk factors, and long-term outcomes of the heart block after treatments by transcatheter pmVSD closure in child patients.

## Patients and methods

The data underlying this article will be shared on reasonable request to the corresponding author.

### Patient population

From June 2002 to June 2020, 1131 consecutive pediatric patients with pmVSD underwent successful transcatheter closure procedure with a single device in our center. Due to incomplete follow-up data for 55 patients, a total of 1076 patients were finally included in the analysis. All patients’ guardians provided written informed consent before participation. The local Ethics Committee of Shandong Provincial Hospital Affiliated to Shandong First Medical University approved the study protocol. This study complied with the ethical standards of the Declaration of Helsinki.

The inclusion criteria were as follows: ages ≥ 2 years or body weight ≥ 10 kg, diagnosed with hemodynamically significant pmVSD (e.g., cardiomegaly on chest X-ray; left atrial enlargement; and LV volume overload), defects identified at 9- to 12-o’clock positions in the short-axis parasternal view by transthoracic echocardiography (TTE), and pulmonary artery systolic pressure < 70 mmHg on TTE.

### Applied devices

The major devices used in this study were the Amplatzer eccentric VSD occluder (AGA Medical Corp., Golden Valley, MN, United States), modified double-disk VSD occluders (Shanghai Shape Memory Alloy, Shanghai, China; Lifetech Scientific, Shenzhen, China; and Starway Medical, Beijing, China), and Amplatzer Duct Occluder II (ADO II) device (St. Jude Medical, St. Paul, MN, United States). There were 3 subtypes of modified double-disk VSD occluders, namely symmetric, eccentric, and thin-waist. Methods of device selection and their descriptions have been reported elsewhere (4). However, depending on the shape of the ventricular defects, we also employed other types of closure devices like patent ductus arteriosus occluder, vascular plug device, and spring coil to close the pmVSD, although in a small number of cases.

### Transcatheter closure procedure

All patients underwent conventional electrocardiography (ECG), chest X-ray, TTE, and 24 h Holter monitoring before the catheterization procedure. The procedure for occluder implantation has been described in detail previously (4). LV angiography (ANG) was performed to estimate the shape, location, and size of the defect, as well as the distance of the defect from the aortic valve. Before releasing the selected device, left cardiac ventriculography, ascending aortography, and TTE were performed to confirm appropriate device position, verify complete closure of the defect, and detect tricuspid or aortic regurgitation and stenosis. After device implantation, TTE was repeated to ensure normal tricuspid and aortic valve function.

### Post-procedure management

All patients received continuous ECG telemetry monitoring until discharge. Chest X-ray and TTE were performed on post-procedure day 1, then ECG was continued once daily throughout hospital stay. All patients underwent 24 h Holter monitoring from day 3 to 5 post-procedure. Furthermore, another 24 h Holter monitoring was performed on patients who developed heart block before discharge. If there were no complications, patients were discharged between day 5 and 7 days after the procedure. A standard oral dose of aspirin (3−5 mg/kg) was prescribed daily for the following 6 months in all patients.

In this study, the diagnostic criteria for postprocedural heart block (i.e., AVB or BBB) were either *de novo* onset of heart block or progression of a pre-existing heart block. If patients developed any of the AVB, CLBBB, or complete right BBB (CRBBB) symptoms, intravenous dexamethasone was administered at a dosage of 0.5−1.0 mg/kg (maximum, 10 mg) daily for 3 to 5 days and then gradually tapered the dose (intravenous dexamethasone or oral prednisone) over 2 weeks.

### Follow-up protocol

The follow-up ECG, chest X-ray, and TTE tests were scheduled for 1, 3, 6, and 12 months after the pmVSD closure and yearly thereafter. Additionally, 24 h Holter monitoring was carried out for each patient who suffered from AVB, CLBBB, or CRBBB at each outpatient visit. The follow-up data were available until December 2021. Patients with at least 1 year of follow-up were enrolled in the study. We characterized the postprocedural heart block as an early-onset (developing within 1 week of procedure), and late-onset (initiating after 1 week of procedure) adverse event. The postprocedural heart block patients who could restore their normal heart conduction by the last follow-up visit were included in the recovery group, and those who failed were allocated to the persistence group.

### Statistical analysis

Independent variables used in the analysis included gender, age, body weight, duration of hospital stay, defect diameter on TTE and ANG (inlet and outlet), subaortic rim (distance between the defect and aortic valve), membranous aneurysms (yes and no), aneurysmal tissue involving the tricuspid valve (yes and no), the left disk placement within the aneurysmal tissue (yes and no), device type (symmetric, eccentric, thin-waist, ADO II, and others), device size, device diameter/defective outlet diameter (measured on ANG) ratio, device diameter/patient’s body weight ratio, procedure time, fluoroscopic time, and radiation dose.

Statistical analysis was performed using the SPSS software v25.0 (SPSS Inc., IL, United States). Data are expressed as counts and/or percentages for categorical variables and mean ± standard deviation (SD) or median with range for continuous variables. Univariate analysis was performed by chi-square (χ*^2^*) test, Fisher’s exact test, Student’s *t*-test, and analysis of variance (ANOVA). Multiple logistic regression analysis was conducted to examine risk factors for the occurrence and persistence of postprocedural heart block. Results with a value of *P* < 0.05 were considered statistically significant. Independent variables with statistical significance in the univariate analysis entered the multivariate model.

## Results

### Heart block after transcatheter closure of perimembranous ventricular septal defect

Data were collected from 1076 patients, aged from 1 year and 8 months to 18 years, with a median follow-up time of 64 months (range:1−19 years). The general characteristics of patients are summarized in Table 1.

**TABLE 1:** General characteristics.

Patients	1076
Sex (F/M)	542 (50.4%)/534 (49.6%)
Age, years	3.6 (1.7–18)
**Age groups**	
<3 years	309 (28.7%)
3–6 years	611 (56.8%)
>6 years	156 (14.5%)
Weight, kg	16 (9–77)
**Weight groups**	
<15 kg	372 (34.6%)
15–20 kg	453 (42.1%)
>20 kg	251 (23.3%)
**Combined procedures**	
PDA closure	9
ASD closure	9
Pulmonary valvuloplasty	1
Balloon dilation of the CoA	1
**Device used**	
Symmetric	703 (65.3%)
Eccentric	149 (13.8%)
Thin-waist	87 (8.1%)
ADO II	131 (12.2%)
Others	6 (0.6%)

Data presented as n (%) or median with rang. PDA, patent ductus arteriosus; ASD, atrial septal defect; CoA, coarctation of aorta; ADO II, Amplatzer Duct Occluder II.

Postprocedural heart block was recorded in 234 patients (21.8%) (Table 2), and the right BBB (RBBB) was the most common type of heart block, accounting for more than half of these cases, followed by left BBB (LBBB) and AVB. In 140 patients (61.1%), the postprocedural heart block developed within 1 day of procedure, and the median interval for the initial occurrence of heart block was 1 day (range: 1 day to 4 years) post-procedure (Table 3). Out of 234 patients, 229 patients exhibited post-procedure early-onset heart block within 1 week, and the remaining 5 cases reported the late-onset subtype occurring between 1 month and 4 years. During the post-procedure hospitalization and follow-up reviews, 138 patients returned to normal heart conduction and were counted in the recovery group. The remaining 96 subjects (8.9%) retained heart block and were included in the persistence group (Table 2). Notably, 8 patients in the recovery group suffered from recurrent heart block (Table 2).

**TABLE 2:** Post-procedural, persistent, and recurrent heart blocks.

	Post-procedure heart blocks, n	Persistent heart blocks, n	Recurrent heart blocks, n
AVB	13	4	0
First-degree AVB	6	0	0
Second-degree AVB	2	1	0
Mobitz type 1	1	1 (Combined IRBBB)	0
Mobitz type 2	1	0	0
Third-degree AVB	5	3	2
RBBB	175	85	0
IRBBB	132	55	1
CRBBB	43	30	0
LBBB	38	6	0
LAH	13	2	1
CLBBB	25	4	3
CRBBB + LAH	8	1	1
Total	234	96	8

AVB, atrioventricular block; RBBB, right bundle branch block; IRBBB, incomplete right bundle branch block; CRBBB, complete right bundle branch block; LBBB, left bundle branch block; CLBBB, complete left bundle branch block; LAH, left anterior hemiblock.

**TABLE 3:** Onset time of post-procedure heart block.

Onset time	Cases, n
1 day	140
2 days	27
3 days	27
4 days	17
5 days	12
6 days	6
1 month	1
6 months	2
2 years	1
4 years	1

Of 13 AVB patients, 5 individuals (0.46%) had cAVB. Their clinical characteristics and follow-up results are shown in Table 4. The preprocedural ECG in 4 cases was normal, except for 1 case with incomplete RBBB (IRBBB). Early-onset cAVB was reported in 3 cases and late-onset in 2 cases. Three patients underwent permanent pacemaker implantations. One patient was reverted to the second degree Mobitz type I AVB, and the other patient suddenly died from cAVB 40 days post-procedure, even after recovering to the CRBBB. The case of death was a 4-year-old boy. LV angiography showed a membranous aneurysm with four outlets, with an inlet diameter of 8.6 mm and the larger outlet diameter of 3 mm. A 6 mm thin-waist occluder was selected, and the left disk was placed at the inlet. Six first-degree and 2 s-degree early-onset AVBs were reported within 5 days of the procedure and were completely reverted to the normal conduction after treatment.

**TABLE 4:** Clinical characteristics and follow-up evaluation of patients with complete atrioventricular block.

Patient	Sex	Age (y)	Weight (kg)	VSD size on angio (mm)	Device type	Device size (mm)	Preprocedural ECG	Onset time post-procedure (days)	Recovery time post-procedure (days)	Temporary pacemaker	Permanent pacemaker	ECG at last follow-up
1	F	3	15	4.9	Eccentric	10	IRBBB	5/446	10/N	No	Yes	Pacing rhythm
2	F	2.8	11.5	8.4	Eccentric	10	Normal	1407	N	No	Yes	Pacing rhythm
3	M	4	15	3.0	Thin-waist	6	Normal	2/40	7/N	Yes	No	Death
4	F	7.5	31	5.5	Eccentric	12	Normal	808	N	No	Yes	Pacing rhythm
5	F	5.7	20.5	5.5	Eccentric	12	Normal	2/31	20/36	Yes	No	Second-degree Mobitz I AVB + IRBBB

VSD, ventricular septal defect; ECG, electrocardiogram; AVB, atrioventricular block; IRBBB, incomplete right bundle branch block.

Among the 25 CLBBB patients, 23 patients developed early-onset symptoms, and the other two cases were late-onset, occurring 6 months post-procedure. Eventually, 21 subjects restored normal sinus rhythm, while 4 subjects continued to have persistent CLBBB. Of the recovered cases, 4 cases showed restoration of normal cardiac conduction after the defect repair and removal of the surgical device on 6, 6, 13, and 40 days post-procedure, respectively. In 4 cases of persistent CLBBB, 3 cases had normal LV end-diastolic diameter (LVEDD) and LV ejection fraction (LVEF) with no clinical symptoms. One case received cardiac resynchronization therapy due to reduced exercise capacity, increased LVEDD, and decreased LVEF (40%) 58 months after the disease onset. Then, both LVEDD and LVEF were returned to the normal in 4 months. All 13 cases of left anterior hemiblock (LAH) were early-onset. The normal conduction was restored in 10 cases, 2 cases had persistent LAH, and 1 case progressed to CRBBB after recovery.

Except for 1 case of CRBBB, 42 cases of CRBBB, and 8 cases of CRBBB with LAH exhibited early-onset symptoms. In the group of 42 CRBBB patients, 18 subjects were able to return to normal heart conduction. While in the group of 8 CRBBB plus LAH patients, only 2 individuals recovered completely, 4 were reverted to CRBBB only, and 1 person temporarily restored the normal conduction but reported recurrent CRBBB events during follow-up, and 1 subject did not recover at all. Furthermore, among 132 IRBBB cases, 77 cases were included in the recovery group, and recurring BBB events were recorded in 1 case.

### Risk factors for the occurrence of heart block

As shown in Table 5, there were significant differences between patients with and without heart block in terms of patient characteristics and the device application. It appeared that lower body weight (*P* = 0.010), use of eccentric or thin-waist occluder (*P* = 0.002), oversized devices (*P* < 0.001), the left disk placed at the inlet (*P* = 0.003), and longer procedural and fluoroscopic times (*P* < 0.001) were significant etiological factors in inducing heart block. Moreover, patients with heart block required a longer hospital stay (*P* = 0.001). Furthermore, comparisons of baseline variables between patients with and without heart block were performed using four main types of devices (Tables 6–9). In patients using symmetric occluder, oversized devices (*P* < 0.001), the left disk placed at the inlet (*P* = 0.021), longer procedural and fluoroscopic times (*P* = 0.022, *P* = 0.047, respectively) were significantly associated with the occurrence of heart block. In patients with eccentric occluder, younger age (*P* = 0.007), lower body weight (*P* = 0.034), larger defect inlet diameter (*P* = 0.029), bigger occluder size (*P* = 0.035), and an increase in the ratio of device diameter to patient’s body weight (*P* < 0.001) were critical determinants of heart block. In patients using a thin-waist occluder, the left disk placed at the inlet (*P* = 0.028), an increase in the ratio of device diameter to patient’s body weight (*P* = 0.005), and longer procedural and fluoroscopic times (*P* = 0.001, *P* = 0.018, respectively) were closely correlated to heart block. There were no significant differences between these variables in patients with ADO II devices.

**TABLE 5:** Risk factors for occurrence of heart block.

	Non-heart block *n* = 842	Heart block *n* = 234	*P*	Multivariate analysis	
				Adjusted OR (95% CI)	*P*
Sex			0.542		
Female	420 (49.9%)	122 (52.1%)			
Male	422 (50.1%)	112 (47.9%)			
Age, years	4.51 ± 2.69	4.20 ± 2.38	0.109		
Weight, kg	18.82 ± 8.99	17.40 ± 6.86	0.010	0.984 (0.947-1.022)	0.407
Hospital stay, d	9.11 ± 2.38	9.92 ± 3.49	0.001	1.087 (1.031-1.146)	0.002
Inlet diameter by TTE, mm	6.90 ± 2.92	6.98 ± 2.72	0.685		
Outlet diameter by TTE, mm	3.71 ± 1.42	3.69 ± 1.42	0.896		
Subaortic rim, mm	2.21 ± 1.51	2.03 ± 1.51	0.104		
Membranous aneurysms			0.179		
No	310 (36.8%)	75 (32.1%)			
Yes	532 (63.2%)	159 (67.9%)			
Aneurysmal tissue involving the tricuspid valve			0.160		
No	461 (54.8%)	116 (49.6%)			
Yes	381 (45.2%)	118 (50.4%)			
The left disk placed within the aneurysmal tissue			0.003		
No	691 (82.1%)	211 (90.2%)			
Yes	151 (17.9%)	23 (9.83%)		0.568 (0.348-0.928)	0.024
Inlet diameter on angio, mm	6.47 ± 3.33	6.77 ± 3.29	0.225		
Outlet diameter on angio, mm	3.35 ± 1.38	3.21 ± 1.26	0.165		
Device type, n			0.002		
Symmetric	574 (68.2%)	129 (55.1%)		Ref.	Ref.
Eccentric	105 (12.5%)	44 (18.8%)		1.312 (0.842-2.045)	0.230
Thin-waist	60 (7.13%)	27 (11.5%)		1.759 (1.023-3.022)	0.041
ADO II and others	103 (12.2%)	34 (14.5%)			
Device diameter, mm	6.38 ± 2.04	6.76 ± 2.32	0.022	1.037 (0.874-1.230)	0.676
Device/defect	2.01 ± 0.49	2.18 ± 0.51	<0.001	1.809 (1.322-2.476)	<0.001
Device/weight	0.39 ± 0.18	0.43 ± 0.18	0.005	1.202 (0.122-11.864)	0.875
Procedure time, min	80.47 ± 36.91	95.35 ± 47.40	<0.001	1.000 (0.994-1.007)	0.944
Fluoroscopic time, min	15.41 ± 13.46	21.32 ± 21.01	<0.001	1.031 (0.012-1.050)	0.001
Radiation dose, mGy	147.77 ± 166.92	167.95 ± 154.10	0.097		

Data presented as n (%) or mean ± standard deviation. OR, odds ratio; CI, confidence interval; ADO II, Amplatzer Duct Occluder II.

**TABLE 6:** Risk factors for occurrence of heart block in patients using symmetric occluder.

	Non-heart block *n* = 574	Heart block *n* = 129	*P*	Multivariate analysis	
				Adjusted OR (95% CI)	*P*
Sex			0.693		
Female	296 (51.6%)	69 (53.5%)			
Male	278 (48.4%)	60 (46.5%)			
Age, years	4.35 ± 2.53	4.34 ± 2.66	0.981		
Weight, kg	18.26 ± 8.30	17.57 ± 7.32	0.382		
Hospital stay, d	9.18 ± 2.38	9.70 ± 3.06	0.072	1.057 (0.982-1.138)	0.138
Inlet diameter by TTE, mm	7.01 ± 2.75	7.04 ± 2.39	0.902		
Outlet diameter by TTE, mm	3.81 ± 1.43	3.69 ± 1.43	0.414		
Subaortic rim, mm	2.36 ± 1.42	2.23 ± 1.33	0.345		
Membranous aneurysms			0.346		
No	180 (31.4%)	35 (27.1%)			
Yes	394 (68.6%)	94 (72.9%)			
Aneurysmal tissue involving the tricuspid valve			0.235		
No	309 (53.8%)	62 (48.1%)			
Yes	265 (46.2%)	67 (51.9%)			
The left disk placed within the aneurysmal tissue			0.021		
No	457 (79.6%)	114 (88.4%)			
Yes	117 (20.4%)	15 (11.6%)		0.531 (0.293-0.960)	0.036
Inlet diameter on angio, mm	6.63 ± 3.00	6.72 ± 2.83	0.737		
Outlet diameter on angio, mm	3.55 ± 1.38	3.19 ± 1.18	0.003	0.975 (0.794-1.197)	0.807
Device diameter, mm	6.51 ± 2.02	6.87 ± 2.35	0.076		
Device/defect	1.92 ± 0.44	2.21 ± 0.54	<0.001	3.253 (2.031-5.212)	<0.001
Device/weight	0.41 ± 0.19	0.44 ± 0.19	0.159		
Procedure time, min	77.52 ± 32.13	85.87 ± 38.08	0.022	1.001 (0.991-1.010)	0.908
Fluoroscopic time, min	14.49 ± 12.02	17.22 ± 14.45	0.047	1.018 (0.994-1.042)	0.141
Radiation dose, mGy	131.36 ± 137.55	139.86 ± 119.27	0.516		

Data presented as n (%) or mean ± standard deviation. OR, odds ratio; CI, confidence interval.

**TABLE 7:** Risk factors for occurrence of heart block in patients using eccentric occluder.

	Non-heart block *n* = 105	Heart block *n* = 44	*P*	Multivariate analysis	
				Adjusted OR (95% CI)	*P*
Sex			0.841		
Female	53 (50.5%)	23 (52.3%)			
Male	52 (49.5%)	21 (47.7%)			
Age, years	5.34 ± 3.50	4.03 ± 2.23	0.007	0.861 (0.603−1.231)	0.413
Weight, kg	21.70 ± 12.12	17.48 ± 7.69	0.034	1.059 (0.951−1.180)	0.294
Hospital stay, d	8.78 ± 2.94	10.61 ± 5.05	0.028	1.157 (1.026−1.305)	0.017
Inlet diameter by TTE, mm	5.20 ± 2.05	5.94 ± 2.33	0.056		
Outlet diameter by TTE, mm	3.81 ± 1.43	3.69 ± 1.43	0.414		
Subaortic rim, mm	1.07 ± 1.64	1.14 ± 1.78	0.810		
Membranous aneurysms			0.090		
No	81 (77.1%)	28 (63.6%)	Ref.		
Yes	24 (22.9%)	16 (36.4%)	0.100		
Aneurysmal tissue involving the tricuspid valve			0.447		
No	91 (86.7%)	36 (81.8%)			
Yes	14 (13.3%)	8 (18.2%)			
Inlet diameter on angio, mm	4.54 ± 2.38	5.51 ± 2.65	0.029	1.029 (0.845−1.254)	0.776
Outlet diameter on angio, mm	3.49 ± 1.63	4.00 ± 1.52	0.078		
Device diameter, mm	7.05 ± 2.52	7.98 ± 2.19	0.035	0.833 (0.602−1.153)	0.833
Device/defect	2.16 ± 0.54	2.10 ± 0.43	0.487		
Device/weight	0.39 ± 0.18	0.51 ± 0.18	<0.001	190.867(1.458−24989.236)	0.035
Procedure time, min	106.69 ± 54.23	117.73 ± 53.13	0.256		
Fluoroscopic time, min	22.67 ± 18.66	29.03 ± 25.98	0.095		
Radiation dose, mGy	225.48 ± 239.05	225.50 ± 213.98	0.981		

Data presented as n (%) or mean ± standard deviation. OR, odds ratio; CI, confidence interval.

**TABLE 8:** Risk factors for occurrence of heart block in patients using thin-waist occluder.

	Non-heart block *n* = 60	Heart block *n* = 27	*P*	Multivariate analysis	
				Adjusted OR (95% CI)	*P*
Sex			0.504		
Female	20 (33.3%)	11 (40.7%)			
Male	40 (66.7%)	16 (59.3%)			
Age, years	5.32 ± 3.15	4.23 ± 1.99	0.105		
Weight, kg	21.31 ± 10.99	17.43 ± 6.23	0.090		
Hospital stay, d	9.07 ± 2.06	10.67 ± 3.80	0.048	1.188 (0.962−1.467)	0.110
Inlet diameter by TTE, mm	10.83 ± 2.53	9.83 ± 3.39	0.131		
Outlet diameter by TTE, mm	3.60 ± 1.21	4.12 ± 1.48	0.090		
Subaortic rim, mm	2.26 ± 1.54	1.89 ± 1.56	0.305		
Membranous aneurysms			1		
No	1 (1.67%)	0 (0.00%)			
Yes	59 (98.3%)	27 (100%)	.		
Aneurysmal tissue involving the tricuspid valve			0.345		
No	2 (3.33%)	3 (11.1%)			
Yes	58 (96.7%)	24 (88.9%)			
The left disk placed within the aneurysmal tissue			0.028		
No	37 (61.7%)	23 (85.2%)			
Yes	23 (38.3%)	4 (14.8%)		0.497 (0.134−1.846)	0.296
Inlet diameter on angio, mm	11.12 ± 2.60	10.89 ± 2.82	0.711		
Outlet diameter on angio, mm	3.15 ± 0.93	3.32 ± 0.99	0.441		
Device diameter, mm	6.03 ± 1.61	6.85 ± 2.09	0.078		
Device/defect	2.00 ± 0.53	2.11 ± 0.54	0.364		
Device/weight	0.33 ± 0.14	0.43 ± 0.17	0.005	30.962(0.675−1421.227)	0.079
Procedure time, min	85.83 ± 36.81	129.82 ± 59.96	0.001	1.012 (0.992−1.032)	0.237
Fluoroscopic time, min	20.50 ± 15.43	36.54 ± 31.84	0.018	1.011 (0.965−1.060)	0.647
Radiation dose, mGy	230.10 ± 256.31	260.26 ± 168.22	0.578		

Data presented as n (%) or mean ± standard deviation. OR, odds ratio; CI, confidence interval.

**TABLE 9:** Risk factors for occurrence of heart block in patients using Amplatzer Duct Occluder II (ADO II).

	Non-heart block *n* = 97	Heart block *n* = 34	*P*	Multivariate Analysis	
				Adjusted OR (95% CI)	*P*
Sex			0.520		
Female	47 (48.5%)	19 (55.9%)			
Male	50 (51.5%)	15 (44.1%)			
Age, years	3.98 ± 1.97	3.84 ± 1.67	0.718		
Weight, kg	17.30 ± 6.59	16.66 ± 4.02	0.594		
Hospital stay, d	9.19 ± 1.91	9.29 ± 1.85	0.774		
Inlet diameter by TTE, mm	5.55 ± 2.40	5.87 ± 2.18	0.499		
Outlet diameter by TTE, mm	2.85 ± 1.10	2.62 ± 0.61	0.252		
Subaortic rim, mm	2.59 ± 1.29	2.51 ± 1.31	0.088		
Membranous aneurysms			0.249		
No	47 (48.5%)	12 (35.3%)			
Yes	50 (51.5%)	22 (64.7%)			
Aneurysmal tissue involving the tricuspid valve			0.182		
No	57 (58.8%)	15 (44.1%)			
Yes	40 (41.2%)	19 (55.9%)			
The left disk placed within the aneurysmal tissue			1		
No	89 (91.8%)	30 (88.2%)			
Yes	8 (8.2%)	4 (11.8%)			
Inlet diameter on angio, mm	4.59 ± 3.37	5.31 ± 3.40	0.283		
Outlet diameter on angio, mm	2.16 ± 0.38	2.15 ± 0.35	0.896		
Device diameter, mm	4.97 ± 0.74	4.71 ± 0.84	0.078		
Device/defect	2.35 ± 0.42	2.23 ± 0.47	0.173		
Device/weight	0.31 ± 0.29	0.29 ± 0.71	0.256		
Procedure time, min	63.25 ± 18.58	75.00 ± 36.24	0.078		
Fluoroscopic time, min	9.26 ± 7.51	14.78 ± 15.83	0.058		
Radiation dose, mGy	106.03 ± 116.04	125.46 ± 118.53	0.405		

Data presented as n (%) or mean ± standard deviation. ADO II, Amplatzer Duct Occluder II; OR, odds ratio; CI, confidence interval.

After adjustment of the confounding factors, multivariate logistic regression analysis revealed that the thin-waist occluder (odds ratio [OR]: 1.759; 95% confidence interval [CI]: 1.023 to 3.022; *P* = 0.041) and oversized device (OR: 1.809; 95% CI: 1.322 to 2.476; *P* < 0.001) were independent risk factors for the occurrence of postprocedural heart block. Besides, heart block was less likely to develop when the left disk of the occluder was placed within the aneurysmal tissue (OR: 0.568; 95% CI: 0.348 to 0.928; *P* = 0.024). Likewise, in patients with symmetric occluders, the oversized occluder (OR: 3.253; 95% CI: 2.031 to 5.212; *P* < 0.001) was confirmed as an independent risk factor for inducing heart block, and the placement of the left disk in the aneurysmal tissue (OR: 0.531; 95% CI: 0.293 to 0.960; *P* = 0.036) was less susceptible to cause heart block. In patients using eccentric occluders, increasing the ratio of device diameter to patient’s body weight (OR: 190.867; 95% CI: 1.458 to 24989.236; *P* = 0.035) was significantly associated with the onset of heart block. Logistic regression analysis showed no variable was significantly associated with the occurrence of heart block in patients using either a thin-waist occluder or ADO II device.

### Risk factors for persistent heart block

Univariate analysis showed that the following variables might be risk factors for the development of postprocedural heart block: lower body weight (*P* = 0.041), larger defect inlet diameter by TTE (*P* = 0.019), larger defect diameter on ANG (*P* = 0.049), bigger occluder size (*P* = 0.030), late time of occurrence (*P* = 0.011), and longer procedural and fluoroscopic times (*P* = 0.009 and *P* = 0.026, respectively). In multivariate analysis, there was no significant parameter to predict the recovery of heart block (Table 10).

**TABLE 10:** Risk factors for persistence of heart block.

	Recovery group *n* = 138	Persistence group *n* = 96	*P*	Multivariate Analysis	
				Adjusted OR (95% CI)	*P*
Sex			0.281		
Female	76 (55.1%)	46 (47.9%)			
Male	62 (44.9%)	50 (52.1%)			
Age, years	4.26 ± 2.36	4.11 ± 2.41	0.644		
Weight, kg	18.10 ± 7.96	16.40 ± 4.74	0.041		
Hospital stay, d	10.12 ± 3.50	9.65 ± 3.48	0.312		
Inlet diameter by TTE, mm	6.64 ± 2.59	7.48 ± 2.84	0.019	0.930 (0.808-1.070)	0.308
Outlet diameter by TTE, mm	3.61 ± 1.39	3.81 ± 1.46	0.294		
Subaortic rim, mm	1.98 ± 1.36	2.10 ± 1.71	0.587		
Membranous aneurysms			0.948		
No	44 (31.9%)	31 (32.3%)			
Yes	94 (68.1%)	65 (67.7%)			
Aneurysmal tissue involving the tricuspid valve			0.875		
No	69 (50.0%)	47 (49.0%)			
Yes	69 (50.0%)	49 (51.0%)			
The left disk placed within the aneurysmal tissue			0.801		
No	125 (90.6%)	86 (89.6%)			
Yes	13 (9.42%)	10 (10.4%)			
Inlet diameter on angio, mm	6.42 ± 3.24	7.28 ± 3.30	0.049	1.005 (0.891-1.133)	0.941
Outlet diameter on angio, mm	3.07 ± 1.20	3.40 ± 1.33	0.049		
Device type, n			0.068		
Symmetric	79 (57.2%)	50 (52.1%)			
Eccentric	20 (14.5%)	24 (25.0%)			
Thin-waist	14 (10.1%)	13 (13.5%)			
ADO II	25 (18.1%)	9 (9.38%)			
Device diameter, mm	6.49 ± 2.30	7.16 ± 2.32	0.030	1.031 (0.851-1.249)	0.758
Device/defect	2.18 ± 0.50	2.19 ± 0.52	0.898		
Device/weight	0.40 ± 0.18	0.47 ± 0.19	0.006	0.120 (0.013-1.125)	0.063
Procedure time, min	88.38 ± 43.11	105.37 ± 51.56	0.009	0.999 (0.988-1.011)	0.886
Fluoroscopic time, min	18.62 ± 17.76	25.19 ± 24.54	0.026	0.986 (0.961-1.012)	0.295
Radiation dose, mGy	157.54 ± 163.00	182.92 ± 139.80	0.216		
Onset time after procedure			0.011		
≤7 days	138 (100.0%)	91 (94.8%)			
>7 days	0 (0.0%)	5 (5.2%)			

Data presented as n (%) or mean ± standard deviation. OR, odds ratio; CI, confidence interval; ADO II, Amplatzer Duct Occluder II.

## Discussion

Studies have shown that the most common complication associated with the transcatheter closure of pmVSD is the heart block, with cAVB being the most serious subtype (2, 4). Recently, a few cases of CLBBB-induced cardiomyopathies following the pmVSD closure have been reported (7, 9). Although RBBB was previously thought to not affect the cardiac size and function, however, it may facilitate the progression to a more serious heart block during the long-term follow-up (10). Presently, there are no effective techniques and methods to completely avoid the occurrence of postprocedural heart block. Nevertheless, correlation analysis of risk factors for the postprocedural heart block after pmVSD closure in pediatric patients is lacking.

In this cohort, the incidence of heart block was 21.8%, of which RBBB accounted for 74.8%. As shown in other studies, IRBBB is the most common type, which may be related to the fact that right bundle branch is very closely located to the endocardium and slender making it vulnerable to damage. Recent findings suggest that the percentage of incidence of early-onset cAVB has decreased from about 3% to 1% (11), which was only 0.46% in our study. A total of 25 patients experienced CLBBB in this study, and the rate of incidence of 2.32% was consistent with previously published data (3, 7). Notably, certain pre-existing or emerging heart blocks may progress to other types of cardiac malfunctions or even aggravate the existing RBBB, LBBB, and first- or second-degree AVB to cAVB (6, 12). Among these cases, there were 3 IRBBB patients, diagnosed by the preprocedural ECG who progressed to severe types of heart block, including 2 patients with CRBBB and 1 patient with cAVB. A previous study showed that patients with CRBBB cum LAH have a higher possibility of developing cAVB following the occluder implantation (6), but no such case could be detected in this study.

Generally, abnormal bundle branch or atrioventricular conduction occurs early after the device implantation. In our cohort, the majority of patients (97.9%) developed early-onset heart block after the defect closure. Although the exact mechanism of heart block remains undefined, various mechanisms might be considered causative. Since the distribution of the cardiac conduction system is adjacent to the pmVSD boundary, the conduction bundle has a high probability of getting injured by the pressure of the catheter or device. Therefore, the most likely mechanism of postprocedural heart block could be the mechanical compression and associated inflammatory edema of the conduction system. In our experience, inhibition of local inflammation and related edema and/or relief of compression was highly beneficial in most (60.3%) of the early-onset heart block patients facilitating the restoration of normal cardiac conduction after the procedure. However, 8 subjects developed recurring heart blocks, even after their initial symptom reversal using corticosteroids. It should be noted that heart block may also occur later after the occluder closure. The present finding revealed that 5 patients developed such late-onset heart block, including 1 case of CRBBB in a 1-month post-procedure, 2 cases of CLBBB in 6 months post-procedure, and 2 cases of cAVB - one in 2 years and another in 4 years after the procedure, respectively. Postprocedural recurrence and late-onset of cAVB and CLBBB have already been reported, showing the cAVB onset as late as 9 years after the closure procedure (6, 7). Accordingly, close observation of the patient’s ECG during the perioperative period and follow-up should be required.

To our knowledge, recurrent and late-onset heart block cases might be more difficult to treat to restore their normal cardiac conductions, such that 5 late-onset cases exhibited persistent heart block and only 2 of 8 recurrent cases were successfully restored (1 of which experienced surgical removal of the occluder). Due to the progressive damage induced by the hyper-activated inflammatory responses as well as continuous friction of the device disk on the septal myocardium, the formation of fibrosis or scar around the implantation area may be another reasonable explanation for the recurrent and late-onset heart blocks. We found that corticosteroid therapy-mediated transient recovery of normal conduction in one late-onset case and one recurrent case might support the notion of postprocedural short-term inflammation around the implantation area. Furthermore, displacement of the occluder or change of device shape may be another causal factor for damaging the conduction bundle. Therefore, during the follow-up, an X-ray examination may be required particularly for such patients. Finally, abnormal development of the conduction tissue may induce permanent cardiac malfunctions, leading to late-onset heart blocks.

The larger ratio of device size to defect size was identified as an independent risk factor for the occurrence of postprocedural heart block in this study. In our cohort, in the cases of membranous aneurysms with multiple outlets, the larger outlet diameter was less than 4 mm in some cases, and different types of occluders were often available. In our experience, the principle of size selection varies for different types of devices in order to avoid oversized devices. The selected device size of symmetric and eccentric occluders was usually 2 to 4 mm larger than the defect diameter on angiography, and that of the ADO II device was usually 1 to 2 mm larger than the defect diameter, whereas the size of thin-waist occlude should generally be selected according to the inlet diameter of the membranous aneurysms. The larger the device used, the wider the scope of the device disk in contact with the interventricular septum, increasing the susceptibility of compression of the conduction bundle. Furthermore, a thin-waist occlude was proved to be an independent predictor of postprocedural heart block. While device waist diameters were the same for both subtypes, the left disk of the thin-waist occlude was relatively larger (4 mm) than that of the symmetric occlude. Hence, a thin-waist occlude might have interfered with the conduction bundle. We identified that another predictor, the placement of the left disk within the aneurysmal tissue, can significantly reduce the occurring events of heart block. Therefore, for VSD with membranous aneurysms, especially when the inlet diameter is larger, the placement of the left disk within the aneurysmal tissue would be more beneficial. It is further recommended that the left disk should be placed inside the aneurysmal tissue as much as possible, even if a larger aneurysmal pmVSD with multiple outlets is closed, using a thin-waist occlude. If the left disk cannot be completely placed into the aneurysmal tissue, partial placement of the left disk may be considered, which may reduce the compression effect of the left disk on the interventricular septum, at least to some extent. Additionally, in patients using eccentric occluders, increasing the ratio of the device diameter to the patient’s body weight was another independent predictor of heart block. For patients with relatively lower body weight who use a larger eccentric occlude, the ECG should be observed more closely after the procedure.

Compared with the symmetric occlude, the use of the eccentric occlude imposed an increased potential risk for postprocedural heart block. Due to the asymmetric structure of the disk, the force point on the left ventricular septal by the eccentric occlude is closer to the region where the conduction bundle mainly passes through. ADO II appears to have a unique advantage in reducing arrhythmias. Several studies have confirmed these observations (13, 14). Nonetheless, ADO II has not been widely applied to pmVSD closure because of its off-label use and device size limitation. In our cases, lower body weight, longer procedural time, and longer fluoroscopic time appeared to be significantly associated with heart block events, while younger age was not, except in the use of eccentric occlude.

Some authors have confirmed that the onset time of postprocedureal heart block is an independent predictor of its persistence. Nevertheless, these similar observations indicated some obvious distinctions. Yang et al. (15) conclude that the early onset of the postprocedural heart block after the VSD closure is more difficult to restore to the normal conduction. Conversely, Wang et al. (7) suggest that the late-onset CLBBB can be less likely recovered normal conduction. Additionally, some studies considered the late cAVB (>30 days) relatively difficult to recover to normal sinus rhythm (6, 16). According to our follow-up study, none of 5 patients with late-onset heart block restored normal conduction. The late-onset heart block might be associated with the persistent heart block. However, it remains uncertain the exact occurrence time period of postprocedural heart blocks which are easy to recover. Moreover, recurring postprocedural heart blocks are also more difficult to recover (6, 7, 16), indicating that this type of heart block might be another risk factor for developing persistent heart block. In our opinion, the lower body weight of children with heart blocks is less likely to recover. Given the immature myocardium in very low-weight children, the conduction bundle may be more vulnerable to damage. It is also notable that a larger defect diameter and device diameter may be risk factors for persistent heart block. On top of that, longer procedural and fluoroscopic times might increase the likelihood of sustained heart block.

Complete AVB is known to be the most serious type of heart block after VSD occlusion. In our study, the parents of one patient with postprocedural cAVB refused the surgical removal of the occlude. After active treatment, the patient temporarily restored the sinus rhythm. Unfortunately, the patient suffered from a recurrent cAVB after discharge, leading to an acute sudden cardiac death. It has already been confirmed that LBBB induces an abnormal LV contraction pattern resulting in LV dysfunction with a decrease in LVEF. Over time, it remains uncertain whether this abnormal contraction pattern could lead to cardiomyopathy. However, the clinical findings strongly support the presence of LBBB-induced cardiomyopathy (17). As the crucial link between conduction abnormalities and LV dysfunction, mechanical dyssynchrony has been proved the major reason for the LBBB-induced cardiomyopathy (17). Of the 4 cases with persistent CLBBB, one patient progressed to LBBB-induced cardiomyopathy, and another 3 cases were asymptomatic with normal LVEDD and LVEF.

High-dose corticosteroids can alleviate acute inflammatory edema and have been shown in this study to achieve complete or partial relief of most early-onset heart blocks (57/84, 67.9%). Interestingly, in our cohort, it appeared that corticosteroid therapy was more effective against the first- and second-degree AVB and CLBBB, but less effective against cAVB and CRBBB. Studies have proved that steroid therapy has an encouraging effect on the recovery of early heart block after VSD closure (5, 7), but its long-term efficacy has not been demonstrated. If the early-onset heart block has not recovered within 2 weeks of corticosteroid therapy, it could be considered essentially ineffective for that type of heart block. We showed that the corticosteroid therapy was ineffective for all recurrent and late-onset heart block patients, except for one case of relapsed cAVB, which reverted to second degree Mobitz type I AVB with IRBBB. Notably, an oral dose of prophylactic steroids for 2 weeks immediately after VSD closure in children can result in an acceptably low incidence of conduction disturbances, but large-scale case-controlled and follow-up studies are lacking to establish this effect (18).

Zuo et al. (5) reported that 3 patients with early cAVB after transcatheter VSD closure did not improve after drug treatment for 2 weeks when they underwent surgical removal of occluders and defect repair. Finally, all of them restored sinus rhythm the day after surgery. Ovaert et al. (19) reported a case study of two children who presented with cAVB 4 days after the VSD closure, and surgical device removal was followed by a rapid and complete recovery of the atrioventricular conduction. A recent study showed that surgical device removal within 10 days followed by VSD closure was performed in 5 patients with cAVB, resulting in resolution of cAVB in 4 cases (16). Bai et al. (6) described the case of one patient with late-onset cAVB, developed 3 years after VSD device closure. Surgery was performed to remove the occlude and repair the defect. However, the patient could not recover to normal atrioventricular conduction. There are almost no related reports about the impact of surgical removal of the device on CLBBB after VSD device closure. In our cases, 4 cases with CLBBB underwent surgical removal of the occlude device together with defect repair 6, 6, 13, and 40 days post-procedure, respectively, and all patients were restored to the normal conduction. Therefore, in cases of cAVB or CLBBB associated with VSD closure, the conduction system might return to normal after the occlude removal. However, whether the conduction system recovers after surgical removal of the occlude is still unknown, particularly for patients with recurrent and late-onset heart blocks. Furthermore, the timing of surgical occlude removal in patients with postprocedural cAVB or CLBBB remains undefined. The rate of recovery of normal conduction after the occlude removal might be closely related to the time of the last occurrence of cAVB or CLBBB and the timing of surgery. Theoretically, the earlier the heart block develops after the VSD closure, the more likely is the recovery to normal conduction after timely surgical removal of the device. Surgery can eventually relieve the compression of the occlude device on the conduction bundle. Thus, early removal of the occlude could be an appropriate approach to treat post-procedural cAVB or CLBBB.

The majority of patients with persistent postprocedural cAVB require permanent pacemaker implantation. As described in a study involving 17 patients with post-VSD closure cAVB, all 8 cases that failed to restore the sinus rhythm were implanted with permanent pacemakers (6). Cardiac resynchronization therapy (CRT) could result in significantly greater improvement in both LV dyssynchrony and LV contractile dysfunction (20). In a recent report on the post-pmVSD closure-associated HF (7), non-responsiveness to medications, and progressive decrease in LVEF to 34%, one patient who presented with postprocedural CLBBB for nearly 10 years received CRT, which increased the LVEF to 55% in 6 months post-treatment. As observed in one patient in our study, ECG signs of cardiac dyssynchrony had disappeared, and LVEF improved from 40% to 60% 4 months post-CRT. Complete or almost complete recovery of cardiac mechanical synchronization and normalization of LV function by CRT may further support the concept of LBBB-induced cardiomyopathy since the treatment of dyssynchrony alone can cure the cardiomyopathy (17).

### Limitations

There are certain limitations to this study. First, it was a single-center experience with a retrospective study. Second, although our study identified risk factors for heart block after transcatheter device closure of pmVSD, the exact mechanism of its occurrence remains unclear. Furthermore, in multivariate logistic regression analysis, no definitive predictors of the continuance of postprocedural heart block were found. Therefore, a multi-center and prospective study with larger sample size is needed to validate these findings.

## Conclusion

With the largest sample size ever known, we report risk factors and long-term follow-up results for pediatric patients with heart block after transcatheter pmVSD closure. The overall incidence rate of heart block following the device closure of pmVSD in children is relatively high, and its outcomes are mostly favorable, as most of them are temporary. However, owing to the recurrence and late-onset heart block complications, more careful monitoring of sinus rhythm is essential during follow-up examinations.

According to our results, thin-waist occluders and oversized devices should be avoided to prevent postprocedural heart block, and eccentric occluders should be carefully selected. The left disk of a device placed into the aneurysmal tissue is recommended.

## Data availability statement

The original contributions presented in the study are included in the article/Supplementary material, further inquiries can be directed to the corresponding author/s.

## Ethics statement

Written informed consent was obtained from the individual(s), and minor(s)’ legal guardian/next of kin, for the publication of any potentially identifiable images or data included in this article.

## Author contributions

DJ and BH designed the study. DJ, SZ, and YZ analyzed and interpreted the patient data of the cases. JL, YY, JW, YW, XY, JZ, and BH contributed to the intellectual content of this manuscript. DJ wrote the manuscript. BH and JZ supervised the study. All authors contributed to the article and approved the submitted version.
